# Minor Operations

**Published:** 1889-12-14

**Authors:** 


### MINOR OPERATIONS.

The last number of the Australian *Medical Journal* to hand contains an article by Mr. J. H. Webb on "Amputation of the Fingers." The little and ring finger of the left hand, says the author, cannot be of very much service, except as an assistance in gripping, for many savage Indians have theirs chopped off as a religious rite, and if this mutilation were of so much consequence as an impediment to their hunting and fishing, it may fairly be presumed that they would have found some other way of appeasing their deity. But, as a rule, the forefinger, if simply rigid, should not be interfered with. Notwithstanding, however, all that Mr. Webb has to say, the question whether or not injured fingers should be removed must depend more on the employment of the person than on aught else. There are certain avocations in which a stiff finger would be prohibitive, and there are others where it would be not less detrimental. As respects *modus operandi*, Mr. Webb regards cutting off the head of the carpal bone, so that the hand may look elegant, "as silly and absurd." Yet this, he says, was recently done by a distinguished London surgeon, "and the hand left will remain, so long as its owner lives, as illustration how a middle finger should not be taken off." The breadth of the hand is spoiled, and its strength weakened, and the adjacent fingers fall together, so as to impede free flexion. Bryant says that in a labouring man it is wise not to remove the head of the metacarpal bone unless diseased, although in the higher classes it is sometimes expedient to take it away to allow the neighbouring fingers to be brought closer together, and thus improve the aspect of the hand. With the same object it is sometimes wise to take off the head of the metacarpal bone of the little or index fingers obliquely away from the median line of the hand. Another British standard author says: "When it is desirable to diminish deformity even at the expense of some utility, the head of the metacarpal bone should be cut off downwards and forwards to prevent a prominence on the dorsum, and care should be taken during the after treatment to keep the fingers parallel, that they may not cross at the tips. We do not think what Mr. Webb advances will alter the practice of British surgeons, especially as there are many persons who would be glad to sacrifice the end of a metacarpal bone to secure a better-looking hand. Personally, we should be inclined to keep the bit of bone, but in such a matter prejudice must be respected.

